# Systematical Identification of the Protective Effect of Danhong Injection and BuChang NaoXinTong Capsules on Transcription Factors in Cerebral Ischemia Mice Brain

**DOI:** 10.1155/2020/5879852

**Published:** 2020-12-14

**Authors:** Jing Xu, Tingting Wang, Feifei Guo, Enhui Ji, Yi Zhang, Hongwei Wu, Shihuan Tang, Junying Wei, Hongjun Yang

**Affiliations:** Institute of Chinese Materia Medica, China Academy of Chinese Medical Sciences, Beijing 100700, China

## Abstract

Cerebral ischemia has led to a high rate of both disability and mortality with massive healthcare costs. Although transcriptional regulation is typically mediated by different combinations of TFs, a combined regulatory unit to synergistically activate transcription has remained unclear in cerebral ischemia, especially in different drug treatments. In this study, TFs alterations after 6 h cerebral ischemic injury and repair were performed by a concatenated tandem array of consensus transcription factor response elements (catTFREs), and vital TFs were obtained by TFs-target imbalanced network. Drug intervention used Danhong injection (DHI) and BNC (BuChang NaoXinTong Capsules), which has been widely prescribed in Chinese herb medicine for the treatment of cerebrovascular and cardiovascular diseases. There were 198 TFs identified after 6 h MCAO operation, and six TFs (Sox2, Smad3, FoxO1, Creb1, Egr,1 and Smad4) were considered as critical TFs in response to cerebral ischemia. Moreover, Smad3 was identified as a hub TF among six vital TFs, and the transcription activity of Smad3 was further verified. These 6 TFs were all reversed by DHI or BNC, indicating different medications may regulate different transcription factors through TF synergy. Moreover, validation results indicated that Smad3 was a putative target TF for DHI and BNC-mediated protection against cerebral ischemia. The observations of the present study provide a fresh understanding of biomolecules and possible new avenues for therapeutic interventions, in addition to the new intervention pattern for different treatments for ischemia stroke.

## 1. Introduction

Cerebral ischemia, a serious neurological disease, has led to a high rate of both disability and mortality with massive healthcare costs [1]. Though remarkable advancements have been made to understand the mechanisms of cerebral ischemia, it is still ambiguous. A large number of evidence has shown that transcription factors (TFs) play an important role in oxidative stress, inflammation, and apoptosis which are associated with ischemia [2–4]. For example, hypoxia-inducible factor-1*α* (HIF-1*α*) is involved in pathologic conditions such as hypoxia or ischemia and leads to severe cerebral injury [5]. Astrocytic N-Myc downstream-regulated gene-2 (NDRG2)) is upregulated after cerebral ischemia and is involved in inflammation [6]. Signal transducers and activators of transcription (STATs) were activated after cerebral ischemia then promoted the expression of several critical proteins that induce brain injury [7]. Oligodendrocyte transcription factor 1 (Olig1) was found to be an important mediator during the differentiation and remyelination after focal cerebral ischemia [8]. Although transcriptional regulation is typically mediated by distinct combinations of TFs, their combined regulatory cues to synergistically activate transcription have remained unclear in cerebral ischemia which indicated that there requires a need for large-scale quantitative profiling of TFs on cerebral ischemia to reveal TFs synergy unit of functionality after drug treatment.

It is prevalent that Chinese herb medicine exhibited advantages of protective effect against cerebral ischemia. Danhong injection (DHI) and BNC (BuChang NaoXinTong Capsules), a Chinese herb medicine, have been widely used for the treatment of cardiovascular and cerebrovascular diseases in clinics. In the previous study, there are 176 components of BNC such as flavonoids, triterpenoid saponins, and phenolic acids identified, and a number of phytochemical constituents of DHI involving flavonoids, quinochalcones, and catechols are identified by using HPLC-MS [9, 10]. BNC and DHI exhibited the brain protection effect; for example, BNC can protect H9c2 cells from oxidative damage through the ERK1/2 signaling pathway [11] and have anti-ischemia efficiency through intervening amino acids metabolon network [12]. DHI prevents I/R-induced brain damage through activating Nrf2/ARE signaling pathway [13]. Although the protective effect and mechanism have been reported, its pharmacological mechanisms, especially the putative regulatory unit of TFs, are still unclear.

Although the low abundance of TFs makes it difficult to analyze the TFs using proteome profiling, the catTFRE method, a DNA construct of tandem TF DNA response elements, is an approach commonly used to identify endogenous TFs at the proteome scale [14]. The catTFRE method was used for large-scale detection of activated TFs. In addition, according to the catTFRE method, there were as many as 400 TFs from a single cell line and a total of 878 TFs from 11 cell types identified, suggesting that this methodology will facilitate discovering a broad perspective of TF activation, repression, and regulatory synergy after cerebral ischemia [15].

In this study, large-scale quantitative profiling and network pharmacology were integrated to reveal the differently expressed TFs responded to cerebral ischemia and TF synergy units after different drug treatments. Furthermore, six vital TFs were screened out, and Smsd3 was considered as critical TFs against cerebral ischemia by medication of BuChang NaoXinTong Capsules (BNC) and Danhong injection (DHI). In addition, TF synergy indicated there is a common TF unit of functionality of different medications. These results provide new details of how TFs activated after cerebral ischemia, as well as insight into a rationally designed combination of drugs with potential synergy.

## 2. Materials and Methods

### 2.1. Animals and Reagents

Male C57BL/6 mice (Charles River Laboratories) were purchased from the Animal Breeding Centre of Beijing Vital River Laboratories Company (Beijing, China). All animals were housed at 22 ± 2°C with a relative humidity of 50 ± 10% and a 12 h light/12 h dark cycle. The animals had free access to water and fodder (Beijing Keaoxieli Co, Ltd.). All experimental animal procedures were approved in accordance with the Guide for the Care and Use of Laboratory Animals published by the US National Institutes of Health (NIH publication No. 85-23, revised 1996) and were approved by the Academy of Chinese Medical Science's Administrative Panel on Laboratory Animal Care.

The concatenated tandem array of consensus transcription factor response element (catTFRE) DNA was synthesized by GenScript (Piscataway, NJ, USA). Biotinylated catTFRE primers were synthesized by Sigma (St. Louis, MO USA). Nuclear extract prep kits were purchased from Thermo Fisher. DHI (batch number: 13011023) was obtained from Shandong Danhong Pharmaceutical Co., Ltd. (Shandong, China). BNC (batch number: Z131117) was provided by the Buchang Pharma Co., Ltd. (Shanxi, China). Deionized water (*R* > 18.2 M*Ω*) used for all experiments was purified by using a Millipore purification system (Billerica, MA, USA). All other chemicals were of analytical grade reagent unless stated otherwise.

### 2.2. MCAO Surgery and Drug Administration

In this study, the intraluminal occlusion using monofilament for the preparation of the permanent middle cerebral artery occlusion (MCAO) model was applied [16]. Briefly, after anesthetized with 1% pentobarbital sodium, a silicone-coated nylon filament was inserted from the left external carotid artery into the internal carotid artery to occlude the origin of the middle cerebral artery. The whole surgery was completed within 10 min, and the rectal temperature of the mice was maintained at 37 ± 0.5°C throughout the surgical procedure. Sham mice were subjected to the same procedures as the abovementioned with the exception of insertion of the nylon filament into the common carotid artery. According to our previous study [17, 18], six hours later after MCAO surgery, the neurological deficit was determined using a modified Neurological Severity Score which is a composite of motor, sensory, and balance tests. The neurologic findings were scored on a five-point scale: a score of 0 indicated no neurological deficit, a score of 1 (failure to extend left forepaw fully) a mild focal neurological deficit, a score of 2 (circling to the left) a moderate focal neurological deficit, and a score of 3 (falling to the left) a severe focal deficit; mice with a score of 4 did not walk spontaneously and had a depressed level of consciousness [19]. Then, the mice were sacrificed, and the brain was divided into five sections immediately (1 mm thickness); then, the slices were immediately stained with 0.5% 2,3,5-triphenyltetrazolium chloride (Sigma, St. Louis, MO, USA) for 15 minutes at 37°C, and numeric images were captured for the quantification of infarct volume. Volume calculation with edema correction was performed blindly using the following formula: 100 × (contralateral hemisphere volume − noninfarct ipsilateral hemisphere volume)/contralateral hemisphere volume [16].

50 mice were randomly divided into five groups: sham operation (Sham, ten), the model group with water treatment (MCAO, ten), MCAO group with DHI treatment (DHI, 5 mL∙kg^−1^∙time^−1^ dosage, ten), MCAO group with BNC treatment (BNC, 440∙mg∙kg^−1^∙time^−1^ dosage, ten), and MCAO group with Ginaton treatment (Ginaton, 5 mL∙kg^−1^∙time^−1^ dosage, ten). BNC were orally administered twice a day for 5 days; DHI and Ginaton were intraperitoneally injected twice a day for a total of 5 days. On the sixth day, one hour after the last oral administration in the morning, cerebral ischemia was carried out by MCAO. DHI and Ginaton were intraperitoneally delivered immediately after the MCAO surgery. The protein of the entire brain was collected for further analysis [17].

### 2.3. catTFRE Pull-Down and Trypsin Digestion

According to the protocols of manuals, nuclear extract prep kits (Thermo Fisher) were employed to obtain the nuclear extracts of the mouse brain. Nuclear extract was mixed with biotinylated DNA which was preimmobilized on Dynabeads. The mixture was mixed with EDTA/EGTA to obtain 1 mM and 200–250 mM salt concentration and then incubated at 4°C for 120 min. The supernatant was abandoned away, and Dynabeads were washed with NETN (100 mM NaCl, 0.5 mM EDTA, 20 mM Tris-HCl, and 0.5% (vol/vol) Nonidet P-40) two times. Then, trypsin was used to digest the beads overnight.

### 2.4. LC-ESI-MS/MS Measurement and Protein Quantifications

Separation for tryptic peptides was carried out from a C18 column (75 *μ*m inner-diameter, 360 *μ*m outer-diameter ×10 cm, 3 *μ*m C18) with a flow rate of 350 nL/min, and LTQ-Orbitrap Velos (Thermo Scientific, USA) equipped with nanospray ion source was employed to analyze. The spray voltages were 1800 V with the ion transfer tube at 350°C. The range of the survey scan was 375–1600 m/z with resolution 60,000 at m/z 400. The 10 most intense peaks with charge state 2 were obtained whereby performing collision-induced dissociation with a normalized collision energy of 35%, activation time of 5 ms, and one microscan, and the intensity threshold was set at 500. The survey scan was acquired in the LTQ normal scan mode.

The identification of proteins was performed through the mouse RefSeq protein database (updated on 11-05-2017) in Proteome Discovery version 1.3 using MASCOT to achieve a false discovery rate (FDR) lower than 1%. It was set to be 20 ppm for precursor and 0.5 Da for the tolerance of product ions. Oxidation (Met) of Acetyl (N terminus) was considered as a variable modification; carbamidomethyl (Cys) was considered as a fixed modification. One missed cleavage site on trypsin was allowed. Transcription factors (TFs) were assigned according to TF class. The results were analyzed through a method of intensity-based absolute quantification (iBAQ-) based protein quantifications by in-house software. Briefly, the number of theoretical peptides were calculated by in silico protein digestion with a PERL script; then, iBAQ intensities were acquired, and all fully tryptic peptides between 6 and 30 amino acids were counted while missed cleavages were neglected.

### 2.5. Gene Ontology and Pathway Enrichment Analysis and Significant TF Identification

To identify the role of differentially expressed TFs, Gene Ontology (GO) such as biological process, molecular functions, and KEGG pathways were performed by DAVID (https://david.ncifcrf.gov/, version 6.8) [20]. A *p* value <0.05 was considered significant for all the enrichment analyses. Based on the DrugBank database (http://www.drugbank.ca/, version: 5.1.6) and the Online Mendelian Inheritance in Man (OMIM) database(http://www.omim.org/, last updated: May 18, 2020) [21, 22], therapeutic targets related to ischemic stroke were collected in Table S1. Protein–protein interaction analysis is increasingly used to discern important disease-associated TFs and targets that may drive aspects of a disease. For the revelation of vital or hub TF response to ischemia stroke, PPI data with high or medium relevance was obtained through String (http://www.string-db.org/, version 11) [23]; then, based on the network parameters (degree, betweeness, and closeness), vital TFs and hub TF were identified.

### 2.6. EMSA and qPCR

The hub TFs and downstream effector were explored through the LightShift chemiluminescent EMSA kit (Pierce Biotechnology) and qPCR to analyze the potential therapeutic targets. The sense strand sequences of the oligonucleotides for the EMSA are as shown in Table S2. Briefly, after the binding process, the mixture was incubated with biotin-labeled DNA probe (10 fmol) for 15 min at room temperature. DNA probe/protein complexes were then separated by 5% native polyacrylamide gel at 100 V for 60 min, followed by transferring to a nylon membrane (GE Healthcare), and visualized by chemiluminescence according to the manufacturer's instructions (GE ImageQuant LAS 4000mini). qPCR was employed to analyze the mRNA value of the downstream effector, and the ΔCt was calculated as ΔCt = (Ct of target gene) − (Ct of actin gene).

### 2.7. Statistical Analysis

Statistical significance was determined by one-way ANOVA followed by Tukey multiple comparison test or Student's *t*-test, which is expressed as mean ± standard deviation with significant *p* values. A value of *p* < 0.05 was considered statistically significant.

## 3. Results

### 3.1. DHI and BNC Exhibited the Protection Effect against Ischemic Stroke

**Figure 1:**
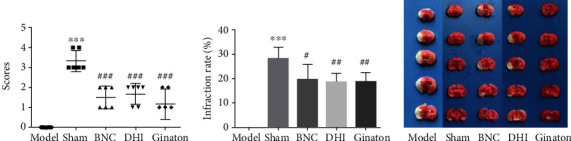
Preventive effect of Ginaton, DHI, and BNC on MCAO mice: neurological score (a), infarct rate (b), and TTC staining of the brain (c). ^∗^*p* < 0.05, ^∗∗^*p* < 0.01, ^∗∗∗^*p* < 0.001, the model group versus the sham group; ^#^*p* < 0.05, ^##^*p* < 0.01, ^###^*p* < 0.001, the BNC group or DHI group or Ginaton group versus the MCAO group.

For the investigation of the BNC and DHI protection against cerebral ischemia, the effects of both drugs were evaluated by Longa's Neurological Severity Score and infarct volume and rate. Based on our previous study, the dose of 5 mL∙kg^−1^∙d^−1^ exhibited beneficial protection [17, 24] and was therefore selected for further studies. The preventive effect of varying dose BNC was assessed, and the medium dose (440 mg∙kg^−1^∙d^−1^) was used to do further studies (Figure S1). As shown in Figure 1(a), after permanent occlusion of the middle cerebral artery, the score in the model group was found to be higher compared with the sham group (*p* < 0.05), indicating prominent ischemic injuries occurred. After the treatment with DHI, BNC, and Ginaton (positive control), the scores were decreased suggesting the neurologic functions were improved in MCAO mice. A similar phenomenon was discovered in infarct volume. As shown in Figures 1(b) and 1(c), mice in the model group showed a higher degree in infarction volume and infarction rate (28.25 ± 4.13), while the DHI, BNC, and Ginaton group were observed to reduce the degree of infarction volume and infarction rate (DHI: 18.55 ± 3.05; BNC: 19.58 ± 5.53; Ginaton: 18.74 ± 3.46, respectively). All these results demonstrated the administration of Ginaton, DHI, and BNC contributes to improving neurologic function and reducing ischemic injures and exhibits a beneficial protective effect against cerebral ischemia.

### 3.2. Alteration of Transcription Factors Activities after Cerebral Ischemia

**Figure 2:**
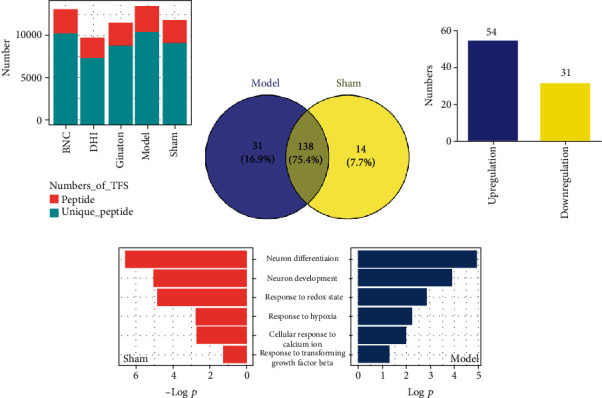
The transcriptional activity of TFs in response to MCAO-induced cerebral ischemia. The number of unique peptides identified of peptides in each group (a). Venn of TFs in the sham and model groups (b). Upregulation and downregulation of differently expressed TFs in the model group compared with the sham group (c). Overlapped items of biological process in both the sham and model groups (d).

Endogenous TFs in the brain after ischemia injury were carried out by the catTFREs method, the tryptic peptides were analyzed on high-resolution Orbitrap MS instruments (Orbitrap Q-Exactive), and protein quantification was performed by intensity-based absolute quantification (iBAQ-) based quantification approach (Figure 2(a)) [25]. Aided by annotation in the mouse RefSeq protein database, there were 198 TFs identified after six hours of MCAO operation. Based on the FOT (fraction of total), the portion of TF expression in all detected TFs in the brain was an indicator of TF abundance.

As shown in Figure 2(b), among the 198 identified activated TFs, there were 183 TFs differently expressed in the sham and model groups and found to provide critical information regarding the clarification of roles of biomolecules behind the diseases. There were 138 TFs activated in both the sham and model groups. In sham mice, there were 154 TFs activated. DAVID 6.8 was employed to investigate the pathway and molecular function of these activated TFs. There were some biological functions involved in brain development such as response to hypoxia, redox state, and negative regulation of neuron differentiation. In model mice, there were 169 TFs activated which responded to the cerebral ischemia. According to the FC (fold change), there were 54 TFs in the ischemic brains which showed a fold change of >1.3, and 31 showed a fold change of <0.7(Figure 2(c)), indicating that numerous TFs may be activated and involved in cerebral ischemia. The annotation results of pathway and biological process or molecular function are obtained from DAVID 6.8. The annotation results indicated that these activated TFs play an important role in various cellular protein processes, such as response to DNA binding, regulation of transcription, and neuron differentiation and development; more importantly, they were found to respond to calcium ion, redox state, and hypoxia indicating these activated TFs were closely related to ischemia. The overlapped GO items between the model and sham are shown in Figure 2(d). After the overlapped biological function and pathway items, response to hypoxia, redox state, transforming growth factor-beta, TGF-beta signaling pathway, and FoxO signaling pathway was found to annotate both in the sham and model groups. Moreover, some potential TF targets reported in the literature were clearly changed. For example, pre-B-cell leukemia homeobox 3 (Pbx3) was downregulated 0.35-fold, and early growth response 1 (EGR1) was upregulated 2.15-fold [2, 26], which implies that the combination of the catTFREs method and iBAQ quantification approach can accurately and quantitatively detect changes in the TF activation and offer an opportunity to find novel potential therapeutic targets.

### 3.3. Alteration of TFs after Treatment with DHI and BNC on TFs against Cerebral Ischemia

**Figure 3:**
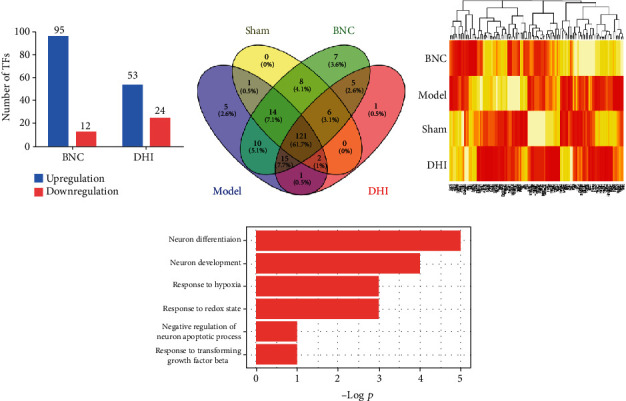
The protective effect of BNC and DHI on transcriptional activity. Upregulation and downregulation of BNC and DHI (a). Venn of TFs in the sham, model, BNC, and DHI groups (b). Hierarchical clustering of the quantitative information of TFs in the four groups (c). The biological process of different expressions TFs in the four groups (d).

In order to investigate the DHI and BNC putative TFs synergy against cerebral ischemia, there were 151 identified in the DHI group and 186 were identified in BNC group. As shown in Figure 3(a), compared with the model group, the BNC treatment group presented 95 TFs with a fold change of >1.3 and 12 TFs with a fold change of <0.7. In the DHI treatment group, there were 53 TFs which exhibited a fold change of >1.3, and 24 showed a fold change of <0.7. As showed in Figure 3(b), among the four group (sham, model, BNC and DHI), 121 TFs among four group were identified both in DHI and BNC group which implied DHI and BNC may intervene in the imbalanced TF work through mediated related TF targets. Based on the FOT of 183 TFs, results were shown by hierarchical clustering (Figure 3(c)). Further, the functions and biological processes of differently expressed TFs were annotated based on DAVID among 121 TFs in four groups; these TFs were involved in neuron differentiation and TGF-beta transforming growth factor production which is related to ischemia (*p* < 0.05, Figure 3(d)).

### 3.4. Potential TFs Synergy of DHI and BNC

**Figure 4:**
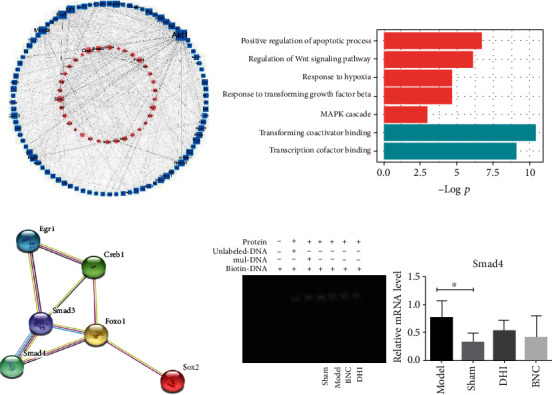
Potential TF synergy of DHI and BNC. TFs-targets imbalanced network related to ischemia (a). GO analysis of six vital TFs (b). PPI network of six vital TFs (c). Verification of Smad3 by EMSA and the value of downstream effector Smad4 mRNA by qPCR (d).

For the identification of vital TFs which responded to cerebral ischemia, important TF-target responses associated with cerebral ischemia were constructed. According to the “network parameter” (degree, betweeness, and closeness), there were 6 TFs (Sox2, Creb1, Smad3, Smad4, Foxo1, and Egr1) screened out as the critical TFs (Figure 4(a)). After GO enrichment analyses, the significant biological function of these six TFs was transcription coactivator binding, transcription cofactor binding, positive regulation of apoptotic processes, response to hypoxia, regulation of Wnt signaling pathway, response to transforming growth factor-beta, and MAPK cascade (Figure 4(b)). Among these six TFs, Egr1 and Smad3 were reversed by DHI and BNC; Sox2, Creb1, and Smad4 were only reversed by DHI; FoxO1 was reversed only by BNC. The result indicated that DHI and BNC can not only exhibit protection to improve neurologic functions and decrease the infarct volume in MCAO mice but also mediate the TF activities to prevent brain ischemia injury.

Further, PPI networks were constructed by retrieving the interaction of these six vital TFs. As detailed in Figure 4(c), the analysis result revealed there was an interaction between these six vital TFs, and Smad3 were identified as hub TFs, indicating that Smad3 and other five TFs may serve as a common regulatory unit of TFs. The interactions between these TFs were reported in the literature, such as Creb1, and were found to bind to endogenous Egr1 promoters [27]; Smad3 and Egr1 interacted via the Smad3 MH2 domain and the EGR1 DNA-binding domain [28]; Creb1 can also bind to the TGFB2 gene promoter in cooperation with Smad3 in glioblastoma [29]; FoxO1 can bind to Smad3 [30]. For further verification of hub TFs to the protective effects of DHI and BNC against cerebral ischemia, EMSA was employed. As shown in Figure 4(d), Smad3 showed a stronger binding in MCAO mice with DHI and BNC treatment. Moreover, the mRNA value of Smad4 was also measured by qPCR. As shown in Figure 4(d), the mRNA value of smad4 which was active downstream effectors of Smad3 showed a similar change in its mRNA expression after cerebral ischemia (*p* < 0.05); although there are no significant changes between the drug treatment and model groups, the mRNA value was showing a decreased trend compared with the model group, after the treatment with DHI and BNC [31].

## 4. Discussion

It is vital for TF regulation of pathogenesis processes in cerebral ischemia. Herein, large-scale quantitative profiling of transcription factors was used to comprehensively explore the transcription factors in MCAO-induced cerebral ischemia. Importantly, the catTFREs method with high-throughput nature, depth of coverage, and high quantitative accuracy can reduce the noise in high-throughput data and reveal the pathogenesis processes in depth of coverage. Pharmacology results indicated that MACO resulted in neurologic function dysfunction, but this injury was relieved by treatment with DHI and BNC. Based on the reliable preventive and protective effect of DHI and BNC against cerebral ischemia, the catTFREs method and network pharmacology were integrated, and six critical TFs were initially screened out. Among these six TFs, Egr1 and Smad3 were reversed by DHI and BNC; the others were reversed by DH or BNC. According to the PPI analysis and literature, Smad3 were identified as hub TFs and interacted with other five TFs, indicating Smad3 served as a hub TF and interacted with other five TFs to respond to ischemia stroke. Further validation of Smad3 reveals that Smad3 were putative targets of BNC and DHI against cerebral ischemia, indicating a different medication may regulate common transcriptional unit through synergy between Smad3 and other TFs.

After cerebral ischemia, a large number of reactive oxygen species and inflammatory cytokines were produced; oxidative and inflammatory injury even apoptosis occurred. Emergence evidence indicated that TFs were significant for the regulation of pathogenesis processes in cerebral ischemia. By making use of the catTFREs method, it offers the opportunity to comprehensively investigate TF function with a low noisy but high throughput and quantity in cerebral ischemia. In this study, six vital TFs were screened out and identified differentially expressed in the sham and model groups, involving various biological processes and molecular functions such as DNA binding, transcription neuron differentiation, and hypoxia (Figure 4(b)). The alteration in these molecules may reveal critical information regarding the clarification of roles of TFs behind the disease. As a member of the early growth response family of Cys 2 His 2-type zinc finger proteins which served as amongst the first pool of genes activated by different physiological stimuli, Egr1 is a sequence-specific transcription factor known to be induced by hypoxia and important in vascular homeostasis [32–34]. Egr1 was found to rapid activation in response to oxygen deprivation underlying ischemic stress [35] and participated in the regulation of inflammation and neuronal damage and resulted in secondary brain damage after cerebral ischemia indicating a variety of downstream genes were activated [36–40]. Egr1 upregulated in MCAO mice in this study which was reported sharply increased after ischemic stroke [26]. Egr1 was enriched in many biological processes, especially hypoxia and ischemia in this study by GO analysis. As a member of the bZIP superfamily, the cAMP response element-binding protein 1 (Creb1) plays a significant role in neural development and neuronal survival. Lacking Creb1 contributes to apoptosis, axonal growth defects, and degeneration of peripheral neurons which suggest the importance of Creb1 in neuronal survival and development [41]. Creb1 could bind to Egr1 promoters [27]. As a member of the bZIP superfamily, the cAMP response element-binding protein 1 (Creb1) was observed improving interaction with Erg1 after hypoxia [42]. Creb1 and Egr1 exhibited a synergistic role in governing MAO-B gene expression under basal and dopamine-induced conditions [43]. Creb1 was decreased in the model group compared with the sham group and also involved in the diverse biological process especially in response to hypoxia and regulation of transforming growth factor-beta production which was one of the TGF-*β* signaling molecules. Transforming growth factor *β* (TGF-*β*) family, including TGF-*β*s and bone morphogenetic proteins (BMPs), serves a pivotal function in the pathogenesis of CNS disorders, especially in cerebral ischemia [44–46]. Smads was activated via BMPs and binds to their specific receptors; then, phosphorylated Smad3 and Smad4 proteins aggregate into complexes, transfer to the nucleus, and react with specific transcription factors or DNA elements to function as transcription factors, thereby regulating the expression of target genes [44]. Herein, Smad3 and Smad 4 were elevated in the model group compared with the sham group, indicating the Smad3 signaling cascade was induced after 6 h ischemia. Forkhead box O1 (FoxO1) belongs to the FoxO subfamily. Growing evidence suggested FoxO1 is critical in cell cycle, apoptosis, energy homeostasis, and regulation of antioxidant genes to exhibit protective effect against oxidative stress, indicating FoxO1 is a vital mediator of oxidative stress [47]. As a member of the Sox family, SRY (sex-determining region Y)-box 2 (Sox2) is expressed at an early stage of central neural system development and marks neural stem cells [48]. Sox2 deficiency causes neurodegeneration and impaired neurogenesis in the adult mouse, such as intervening Alzheimer's disease development by decreasing the *β*-amyloid precursor protein [49], protecting neurons from excitotoxicity by BnNOS-Sox2-Shh axis [50, 51]. Based on the KEGG pathway and GO enrichment analysis, 6 critical TFs were more frequently involved in the BMP signaling pathway (Egr1 and Smad4), FoxO signaling (Smad4, Smad3, and FoxO1), transforming TGF-*β* production, and TGF-*β* signaling (creb1, Smad4, and Smad3), which were all important in pathological processes of cerebral ischemia.

In order to test the effectiveness of our strategy, DHI and BNC were both used to reveal critical TFs in response to MCAO-induced cerebral ischemia. In this study, MCAO was performed to confirm the preventive and protective effect of DHI and BNC in reduced neuron injury. After the catTFREs method and the intensity-based absolute quantification (iBAQ-) based quantification approach analysis, there were 186 TFs activated in BNC and 151 TFs in the DHI group. Consistent with the results from DHI, the enrichment results implied BNC mediated TFs related to the biological process of response to hypoxia, transforming the TGF-*β* production and the pathway of FoxO1 signaling. Although the BNC and DHI may intervene in some similar biological processes and pathways, they may play a different role in some TFs. Sox2 was found to be interacting with Smad3, and Sox2 loss antagonized TGF-*β*1 signaling by interacting with Smad3 in vivo [52]. Creb1, related to response to hypoxia, can cooperate with Smad3 to mediate a maximal induction of Smad7 transcription following stimulation by TGF-*β*3 [53, 54]. FoxO1, only reversed by BNC, serves as the target of TGF-*β*1/Smad3 to promote hepatic gluconeogenesis [55]. The analysis showed that DHI and BNC may intervene in different targets of some similar cellular processes indicating they both have a common intervention pattern.

## 5. Conclusions

In this study, large-scale quantitative profiling and network pharmacology were used to construct the imbalanced network of TFs after cerebral ischemia and critical TFs against cerebral ischemia by medication, BuChang NaoXinTong Capsules (BNC), and Danhong injection (DHI). The in-depth and high quantitative accuracy of the catTFREs method analysis makes it possible and highly valuable for the investigation of TFs alteration response to cerebral ischemia. In this study, this stratagem was used to investigate the TF alteration responded to cerebral ischemia and common transcriptional regulatory of different drugs, DHI and BNC, which exhibited preventive effect in the pharmacology experiment. After investigation of the six key TFs by network pharmacology, Smad3 were found to be the hub TFs connected to other five TFs and constructed the common unit of TFs of DHI and BNC. Further validation indicated that Smad3 and other five TFs are putative target TFs for DHI and BNC against cerebral ischemia. The observations of the present study provide a novel understanding regarding the regulatory mechanism of cerebral ischemic injury, and the common unit of transcriptional regulation found in this study may result in the discovery of novel candidates for the surveillance of cerebral ischemic injury.

## Data Availability

Please contact the corresponding author to access the data supporting this study.

## Supplementary Materials

Supplementary Materials Figure S1. Protective effect of different dose BNC on MCAO mice. Neurological score **(A)**; infarct rate **(B)** and TTC staining of the brain **(C)**. ^∗^*p* < 0.05, ^∗∗^*p* < 0.01, ^∗∗∗^*p* < 0.001, the model group versus the sham group; ^#^*p* < 0.05, ^##^*p* < 0.01, ^###^*p* < 0.001, the BNC group or DHI group or Ginaton group versus the MCAO group. Tables S1 and S2 are the data for the manuscript.
