# Supplementary material for: Systematical Identification of the Protective Effect of Danhong Injection and BuChang NaoXinTong Capsules on Transcription Factors in Cerebral Ischemia Mice Brain
Source: Oxid Med Cell Longev. 2020 Dec 14;2020:5879852. doi: 10.1155/2020/5879852 (PMC7755463; doi:10.1155/2020/5879852)
Supplement: Supplementary Materials — Figure S1. Protective effect of different dose BNC on MCAO mice. Neurological score (A); infarct rate (B) and TTC staining of the brain (C). ∗p < 0.05, ∗∗p < 0.01, ∗∗∗p < 0.001, the model group versus the sham group; #p < 0.05, ##p < 0.01, ###p < 0.001, the BNC group or DHI group or Ginaton group versus the MCAO group. Tables S1 and S2 are the data for the manuscript. [file 5879852.f1.zip › 5879852.f1/Figure S1.docx]

For investigation the different dose BNC protection against ischemia stroke, Longa’s Neurological Severity Score and infarct volume and rate were used to investigate the protective effect of different dose BNC. Compared with sham group, the neurological scores and infarction rate were evidently elevated in model group, indicating a neurological dysfunction after MCAO. After BNC treatment, the BNC-H (880 mg∙kg^-1^∙d^-1^), BNC-M (440 mg∙kg^-1^∙d^-1^) and BNC-L (220 mg∙kg^-1^∙d^-1^) showed significant improvement in neurological scores (Figure S1A). Although the infarction rate was showed reduced trend in different dose BNC group, the medium dose of BNC showed significant decreased level, as detailed in Figure 2B and 2C. Therefore, the medium dose of BNC was used to do further study.


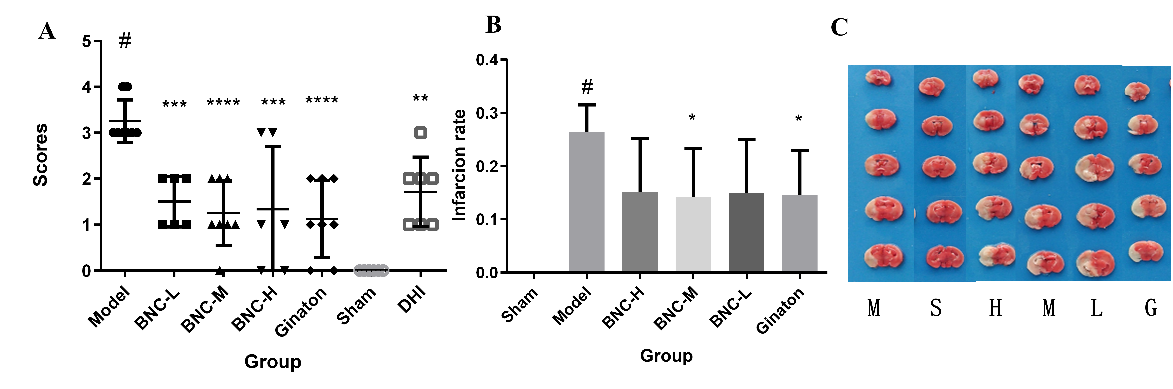


**Figure S1.** Protective effect of different dose BNC on MCAO mice. neurological score **(A)**; infarct rate **(B)** and TTC staining of brain **(C)**. * *p* ＜ 0.05, ** p ＜ 0.01, *** *p* ＜ 0.001, the model group versus the sham group; # *p* ＜ 0.05, ## *p* ＜ 0.01, ### *p* ＜ 0.001, the BNC group or DHI group or Ginaton group versus the MCAO group.
